# Spotlight on global health research

**DOI:** 10.1093/inthealth/ihaa082

**Published:** 2020-11-09

**Authors:** David S Lawrence, Margaret Gyapong

**Affiliations:** Department of Clinical Research, Faculty of Infectious and Tropical Diseases, London School of Hygiene and Tropical Medicine, London, UK; Botswana Harvard AIDS Institute Partnership, Gaborone, Botswana; Institute of Health Research, University of Health and Allied Sciences, Ho, Ghana

Global health research is a discipline in which it is highly possible to cause more harm than good. Universally, the conduct of ethical research is bound by international principles and guidelines and its design and implementation are interrogated by funders and institutional review boards. Research in resource-limited settings is no different in this respect but poses additional ethical considerations due to the nature that the research is conducted alongside or within poorly resourced healthcare systems. The aim of this special issue is to identify work that acknowledges this complexity but demonstrates best practice in the pursuit of fair and equitable approaches to global health research. We were thrilled to receive a total of 27 paper proposals from a broad range of institutions, research teams and geographical locations. After some tough deliberation we are pleased to present the final 12 manuscripts that make up this special issue.

We begin in post-Ebola Sierra Leone, where Peña-Fernández et al.^1^ outline the experience of setting up a transnational research partnership to deliver a parasitology training programme and demonstrate the complexity of forming equitable and ethical research partnerships. Their team highlight that whether something is ethical (or not) cannot be determined simply in an ethics committee meeting. Wright^2^ echoes this sentiment in a short communication summarising a recently published Nuffield Council on Bioethics report: ‘Research in global health emergencies’, arguing that research can only be ethical if it encompasses three core values: equal respect, fairness and helping to reduce suffering, all of which are the responsibility of all the stakeholders or ‘duty bearers’ involved in research. Transnational research partnerships are then scrutinised more broadly under the lens of the Decolonising Global Health movement by Lawrence and Hirsch,^3^ who focus particularly on what researchers from high-income settings can and need to do to make partnerships more equitable.

The design of global health research raises particular ethical issues related to both participant reimbursement and the provision of ancillary care to individuals who may live in resource-limited settings with weak healthcare infrastructure. Reflecting on their ethnographic community-based study of air pollution in Malawi, Saleh et al.^4^ demonstrate the complexity of decision-making around participant compensation and encourage researchers to engage with research participants and communities to develop and evolve their approach. Sansom and colleagues from the Oxford University Clinical Research Unit in Vietnam outline the steps their institution took to develop a fair and transparent research participant compensation and reimbursement framework, encouraging others to learn from and adapt their method.^5^ In their research with ethics committee members and research investigators in Uganda, Ssali et al.^6^ identify shared concerns about the potential for participants to consent to a study as a surrogate for routine healthcare provision, and Nkosi et al.^7^ describe the dilemmas faced by HIV prevention research workers in South Africa when trying to meet the ancillary care needs of their vulnerable participants.

What it means to be vulnerable and the importance of including a broad range of communities in clinical trials is the focus of the research conducted by Khirikoekkong et al.^8^ on the Thai-Myanmar border. The authors show us how and why the design of clinical trials must be adapted to enable vulnerable communities to participate. We also learn from Ngwenya et al.^9^ about how changes in the focus of research in South Africa, in their case from infectious to non-communicable diseases and increasingly towards genetic analyses, should lead to changes in the way we communicate with potential participants to ensure that consent is truly informed and voluntary.

One key message to take home from this special issue is that the social sciences have an immense amount to contribute to global health research. Peay et al.^10^ show us how social science and community engagement performed alongside a HIV cure trial in Thailand helped to determine what is truly ethical, particularly in a dynamic research discipline where the standard of care is rapidly changing. Lees and Enria's comparative ethnographies of preventive clinical trials conducted in Sierra Leone and Tanzania highlight the contributions of critical anthropological engagement in research, taking into account global and local power dynamics and demonstrating the true value of anthropology in clinical trials.^11^ Finally, Henderson et al.^12^ point out that observational studies are also worthy of qualitative enquiry, outlining the bioethical nuances of a cohort study in Thailand.

Thank you to all those who submitted paper proposals, the authors of the final manuscripts and to our reviewers, who kindly gave up their valuable time and used their expertise to improve the quality of this collection. Each of these individual papers is excellent and worth reading but it is only when read together as a combined whole that their true value emerges. When this special issue was conceived we set out with the aim of stimulating discussion around the ethos of global health research, to deepen our understanding of what constitutes responsible conduct in our discipline and to propose areas for improvement. We are sure that after reading this special issue you will be motivated to reflect on the way in which global health research is conducted. We hope this collection will help us to learn from one another as we strive to improve health worldwide.

## Data Availability

None.
