# Corrigendum to: Spotlight on global health research

**DOI:** 10.1093/inthealth/ihaa100

**Published:** 2020-12-10

In the originally published version of this article, the reference list was omitted. This has since been updated to include the reference list.

